# Beef tallow injection matrix for serial crystallography

**DOI:** 10.1038/s41598-021-04714-6

**Published:** 2022-01-13

**Authors:** Ki Hyun Nam

**Affiliations:** 1 Department of Life Sciences, Pohang University of Science and Technology, Pohang, Gyeongbuk 37673 Korea; 2 POSTECH Biotech Center, Pohang University of Science and Technology, Pohang, Gyeongbuk 37673 Korea

**Keywords:** X-ray crystallography, Nanocrystallography

## Abstract

Serial crystallography (SX) enables the visualization of the time-resolved molecular dynamics of macromolecular structures at room temperature while minimizing radiation damage. In SX experiments, the delivery of a large number of crystals into an X-ray interaction point in a serial and stable manner is key. Sample delivery using viscous medium maintains the stable injection stream at low flow rates, markedly reducing sample consumption compared with that of a liquid jet injector and is widely applied in SX experiments with low repetition rates. As the sample properties and experimental environment can affect the stability of the injection stream of a viscous medium, it is important to develop sample delivery media with various characteristics to optimize the experimental environment. In this study, a beef tallow injection matrix possessing a higher melting temperature than previously reported fat-based shortening and lard media was introduced as a sample delivery medium and applied to SX. Beef tallow was prepared by heat treating fats from cattle, followed by the removal of soluble impurities from the extract by phase separation. Beef tallow exhibited a very stable injection stream at room temperature and a flow rate of < 10 nL/min. The room-temperature structures of lysozyme and glucose isomerase embedded in beef tallow were successfully determined at 1.55 and 1.60 Å, respectively. The background scattering of beef tallow was higher than that of previously reported fat-based shortening and lard media but negligible for data processing. In conclusion, the beef tallow matrix can be employed for sample delivery in SX experiments conducted at temperatures exceeding room temperature.

## Introduction

Serial crystallography (SX) is a technique that enables the observation of room temperature structures of various molecules, ranging from macromolecules to small molecules^1–4^. Unlike conventional X-ray crystallography techniques, SX uses intense X-ray pulses or short X-ray exposure, thus reducing radiation damage to crystal samples^5–7^. Moreover, time-resolved chemical and biological reactions of target samples can be observed using pump-probes with photoactivation or liquid composed of a substrate or inhibitor^8–13^. Thus, the SX technique is a very useful tool for observing the structural flexibility and molecular dynamics of molecules at room temperature^2, 5, 14^.

For SX data collection, serial and stable crystal delivery to the X-ray interaction position is crucial^15, 16^. Various sample delivery methods, such as injector^17–22^, syringe^23–25^, fixed-target scanning^26–36^, conveyor belt^8^, capillary^37, 38^ and microfluidics^39–43^, have been developed. Among them, the liquid jet injection method has been widely used since the beginning of SX applications in research; this method is advantageous because it maintains a hydrated environment and a narrow diameter of the injection stream, resulting in very low background scattering^17^. However, this sample delivery method requires a high flow rate to continuously and stably deliver the sample to the X-ray interaction point; thus, in an X-ray free-electron laser facility with a low repetition rate or synchrotron, the number of unexposed crystals is overwhelmingly larger than that of crystals exposed to X-rays, leading to high sample consumption^16, 18, 23^. Therefore, sample delivery at an appropriate flow rate, depending on the characteristics of the X-ray free-electron laser facility or synchrotron, is important for reducing sample consumption^3, 20^.

As an alternative sample delivery method, sample crystals can be embedded in viscous medium and delivered through an injector or syringe^18, 23, 24^. Sample delivery using viscous media maintains stable injection streams, even at low flow rates, because of the viscosity of the substance^16^. Thus, this method is widely applied in X-ray free-electron laser facilities with low repetition or synchrotron X-rays, as it can markedly reduce sample consumption compared to the liquid jet injection method.

To date, lipidic cubic phase (LCP) and hydrophobic and hydrophilic delivery media have been developed and applied in SX data collection^16^. Aliphatic LCP (e.g. monoolein), which has been widely applied as a delivery medium for not only membrane proteins but also water-soluble proteins, generates a stable stream even at very low flow rates^18, 44^. However, this medium may generate an unstable injection stream under ammonium sulfate conditions and alter the phases because of the sample environment such as temperature and humidity^37, 45^. In hydrophilic delivery media, sugar-based [agarose^46^, hyaluronic acid^47^, hydroxyethyl cellulose^48^, sodium carboxymethyl cellulose^49^, wheat starch^50^ and alginate^50^], and polymer-based media [Pluronic F-127^49^, poly(ethylene oxide)^51^, and polyacrylamide^52^] have been developed, which offer the advantage of very low background scattering but often generate unstable streams due to changes in viscosity depending on the crystallization solution^46, 52^. In hydrophobic delivery media, grease^23, 53^, shortening^54^, and lard^55^ have been developed, leading to a stable injection stream; however, background scattering occurs to some degree, although this is not critical for data processing in applied SX data collection. Among these, shortening media can easily control the solid and liquid phases depending on the temperature, making experiment handling convenient and cost-effective^54^. In a previous study, although the melting temperatures of two types of shortening media used for SX were higher than room temperature, the viscosity was decreased by heat from the beamline equipment used in the test hutch, thereby generating an unstable injection stream; eventually, a 20 °C environment was used to generate a stable injection stream for SX data collection^54^. Therefore, changes in the viscosity of the shortening injection medium due to temperature fluctuations may be disadvantageous. To maintain the advantages of shortening and compensate for the disadvantages, lard was used for sample delivery medium for SX data collection because of its higher melting point compared to that of shortening^55^. Lard generates a stable injection stream at room temperature, with similar or lesser background scattering compared to LCP or shortenings^55^.

The room-temperature macromolecular structures generated by SX experiments are biologically more meaningful for observing structural flexibility than data collected in a cryogenic environment in traditional X-ray crystallography^1, 56^. Additionally, many proteins and enzymes show maximum enzyme activity at higher than room temperature^57, 58^. Accordingly, to better understand protein function, it is desirable to observe the crystal structure at the optimum temperature for protein activity. For such research, it is very important to develop a crystal delivery medium that is stable even at higher temperatures compared to previously developed sample delivery media.

In nature, various fat-based substances are similar to shortening and lard; of these, beef tallow and mutton tallow have physical properties similar to those of lard but with higher melting temperatures^59, 60^. As the composition and characteristics of tallow are very similar to those of lard, it may be applied in SX experiments^61^. However, experimental evidence supporting the use of tallow in SX experiments and the inherent characteristics of injection streams and background scattering created are not available.

Thus, in this study, the properties of beef tallow were characterized, and its application as a sample delivery medium for SX experiments was demonstrated. This viscous medium produced very stable injection streams at low flow rates. These results indicate that beef tallow medium can be applied in SX.

## Results

### Preparation and injection of beef tallow injection matrix

In this study, the fat around the ribs of cattle were directly extracted and used as a sample delivery medium. Beef tallow and other soluble substances were obtained from cattle using a heat treatment method widely used in general food science^59^. As unwanted soluble substances may have a non-specific effect on the crystal sample, they were removed from the extracted beef tallow using the phase separation method (Supplementary Fig. S1), as described previously^55^.

In this experiment, > 20 mL of beef tallow was obtained from approximately 100 g of wasted beef tissue. However, this output is likely to vary depending on the fat content of the tissue part used and the refining process.

**Figure 1:**
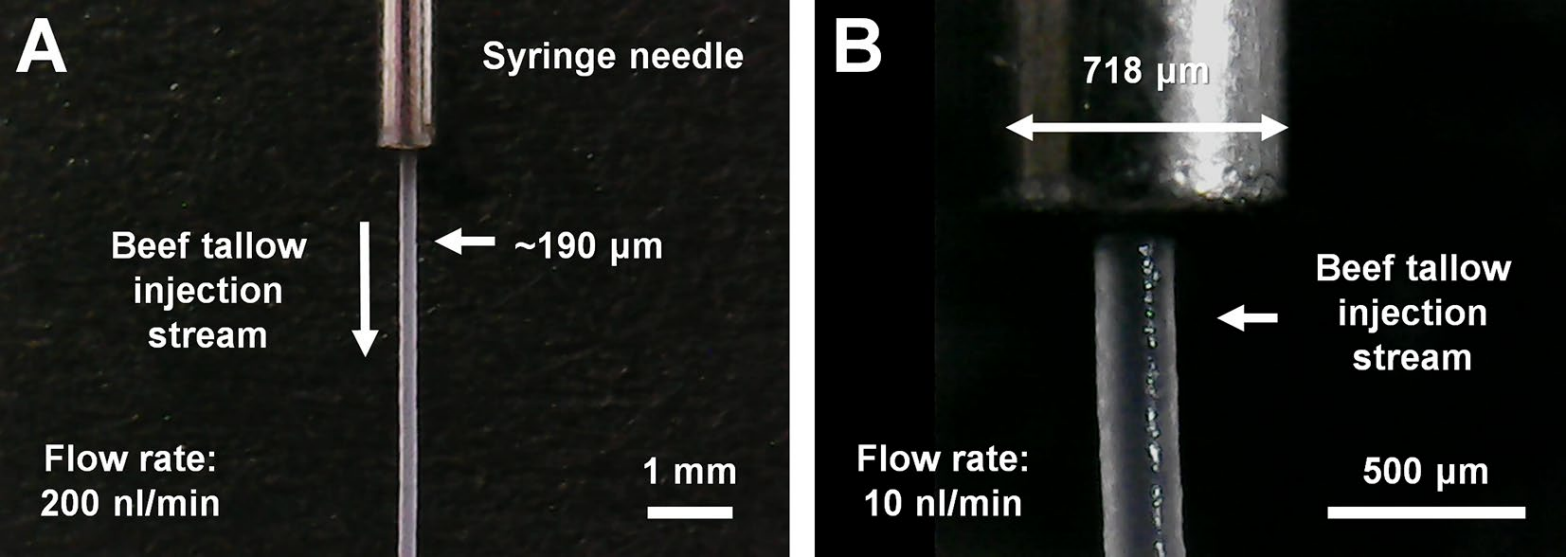
Beef tallow injection stream with embedded lysozyme crystals at flow rates of (**A**) 200 nL/min and (**B**) 10 nL/min. The inner diameters of the syringe needle and the injection stream of the beef tallow were 168 and < 200 μm, respectively.

The melting temperature of beef tallow was visually estimated to be approximately 42 °C. To verify whether the purified beef tallow can be used as a sample delivery medium for SX, an injection experiment was performed using a syringe. Solidified beef tallow in a glass vial was immersed in hot water (< 100 °C) to prepare a liquid-phase, and then transferred to a syringe using a pipette. After the solidification of the beef tallow in the syringe, the syringe was installed vertically in a syringe pump; the sample was extruded through a syringe needle with an inner diameter of 168 μm. At low flow rates of < 200 nL/min, the initial extrusion of the beef tallow delivery medium was curled at the tip of the syringe needle. This problem was overcome as previously reported for sample delivery with shortening^54^, in which the flow rate was initially 2–3 μL/min for approximately 3 s and then stopped to create a stream without curling, which was directed downwards by gravity. The flow rate was lowered, and the beef tallow produced a very stable injection stream (Fig. 1A and Supplementary Videos 1, 2, 3, 4, 5). Remarkably, the beef tallow injection matrix was very stable even at flow rates of 10 nL/min (Fig. 1B). The matrix also produced highly stable injection streams at flow rates of 50, 100, and 200 nL/min (Supplementary Videos 1, 2, 3, 4). The diameter of the injection stream of the beef tallow was approximately 190 μm (Fig. 1), which is wider than the inner diameter of the syringe needle. This phenomenon is also exhibited by other viscous substances, such as polyacrylamide^24^, wheat starch^50^ and alginate^50^, and is hypothesized to occur owing to the characteristics of the viscous material and the blunt-type syringe needle tip. However, this phenomenon does not appear to influence the diffraction of the crystal sample. Furthermore, as the temperature increased beyond the melting temperature, the viscosity of beef tallow reduced and the injection stream became unstable. Additionally, when the viscosity becomes negligible at elevated temperatures, the injected beef tallow forms a drop at the tip of the syringe needle. Purified beef tallow was stored at 4 °C, and no problems were encountered upon using it as an injection matrix at the end of 1 year.

Crystal samples can be grown in crystallization solutions of various chemical compositions. The effect of the crystallization solution on the viscosity of beef tallow was investigated. When beef tallow mixed with salt (final 1 M NaCl or 0.9 M (NH_4_)_2_SO_4_) or polyethylene glycol (PEG) (final 20% [v/v] PEG400, 10% [w/v] of PEG3350, or 10% [w/v] PEG8000), injection media provided a stable stream without the influence of viscosity. In contrast, when beef tallow were mixed with final 20% (v/v) 2-methyl-2,4-pentanediol (MPD), the injection media had low viscosity and provide curled injection stream. Furthermore, to verify the applicability of beef tallow to time-resolved studies using optical light, its absorbance was measured using ultraviolet/visible (UV/vis) spectroscopy. Beef tallow showed an absorption profile in the broad range of 300–700 nm, which tended to increase with increasing thickness (Supplementary Fig. S2).

### SX data collection using beef tallow injection matrix

Although beef tallow provided a very stable injection stream, SX data collection and structural analysis were required to verify whether the extracted material physically affected the crystal sample and if the crystal diffraction intensity was unaffected. To evaluate beef tallow as a sample delivery medium for SX experiments, SX data were collected at room temperature using lysozyme and glucose isomerase crystals as model samples. The crystal suspension containing minimal crystallization solution was gently mixed with beef tallow in a dual syringe setup. Beef tallow is a semi-solid that crumbles readily, similar to previously reported shortenings, lard, and LCP. After mixing the crystals with the beef tallow, no significant physical damage was observed on the surface of the crystals, and the intensity of X-ray diffraction remained unaltered (see below). To verify whether the crystals were stable in beef tallow over the long-term, the embedded crystals were allowed to stand at room temperature for 2 h before conducting SX experiments. Thereafter, the crystals embedded in beef tallow were extruded to the X-ray interaction point through the syringe needle with an inner diameter of 168 μm using a syringe pump. The crystals embedded in the beef tallow were exposed to X-rays for 100 ms, and data were collected at room temperature (Table 1). For each lysozyme and glucose isomerase crystal embedded in beef tallow, 80,000 images were collected in 2.5 h.

**Table 1:** Data collection and refinement statistics for lysozyme and glucose isomerase delivered in beef tallow injection matrix.

Data collection	Lysozyme	Glucose isomerase
Diffraction source	11C beamline, PLS-II	11C beamline, PLS-II
Wavelength (Å)	0.9794	0.9794
Temperature (K)	298.5	298.5
Detector	Pilatus 6 M	Pilatus 6 M
Collected images	80,000	80,000
Hit images	21,922	27,380
Indexed images	20,234	7795
Indexed pattern	23,090	7976
Space group	P4_3_2_1_2	I222
*a*, *b*, *c* (Å)	79.45, 79.45, 38.47	94.33, 99.92, 103.31
α, β, γ (°)	90, 90, 90	90, 90, 90
Resolution range (Å)	80.00–1.55 (1.60–1.55)	72.46–1.60 (1.66–1.60)
No. of unique reflections	18,428 (1809)	64,497 (6368)
Completeness (%)	100.0 (100.0)	100.0 (100.0)
Redundancy	1257.8 (667.4)	250.9 (167.6)
SNR	10.63 (1.87)	4.79 (1.52)
CC	0.9941 (0.6243)	0.9656 (0.5348)
CC*	0.9985 (0.8767)	0.9912 (0.8348)
*R*_split_	6.65 (58.61)	16.70 (70.82)
Wilson *B* factor (Å^2^)	27.02	22.61
**Refinement**		
Resolution range (Å)	56.14–1.55 (1.60–1.55)	71.80–1.80 (1.66–1.60)
*R*_work_	0.1678 (0.2705)	0.1681 (0.2870)
*R*_free_	0.1874 (0.3274)	0.1862 (0.2930)
R.m.s. deviations		
Bonds (Å)	0.013	0.007
Angles (°)	1.519	0.914
**Average *B* factors (Å**^**2**^**)**		
Protein	28.80	21.78
Ligand	33.87	11.72
Water	38.04	33.89
**Ramachandran plot**		
Most favoured (%)	98.43	96.34
Allowed (%)	1.57	3.40
Outliers (%)	0.00	0.26

Values for the outer shell are given in parentheses.

R_split_ = $$\left( {{\raise0.7ex\hbox{$1$} \!\mathord{\left/ {\vphantom {1 {\sqrt 2 }}}\right.\kern-\nulldelimiterspace} \!\lower0.7ex\hbox{${\sqrt 2 }$}}} \right) \cdot \frac{{\mathop \sum \nolimits_{hkl} \left| {I_{hkl}^{even} - I_{hkl}^{odd} } \right|}}{{\frac{1}{2}\left| {I_{hkl}^{even} - I_{hkl}^{odd} } \right|}}$$.

R_free_ was calculated as R_work_ using a randomly selected subset (10%) of unique reflections not used for structure refinement.

For lysozyme, 21,922 images included Bragg peaks, and the X-ray hit rate was 27.40%. Among these, 20,234 images were indexed and 2856 images exhibited multi crystal hits. The indexing rate and multi crystal hit rate were 92.30% and 14.11%, respectively. Data were processed up to 1.55 Å with 100% completeness, including multiple crystal diffraction patterns. The signal-to-noise ratio (SNR), R_split_, correlation coefficient (CC), and CC* of the total data collected were 10.63, 6.65, 0.9941, and 0.9985, respectively (Table 1).

**Figure 2:**
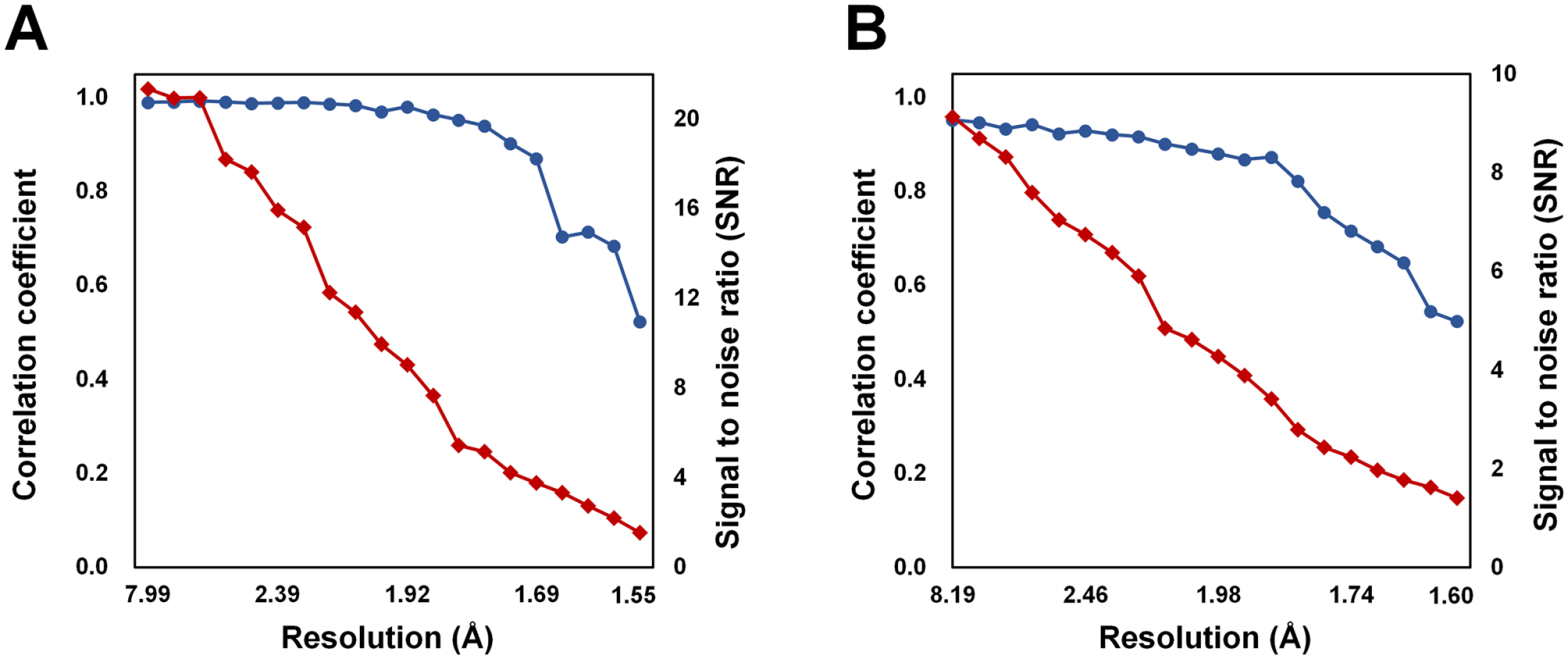
Quality of dataset for (**A**) lysozyme (indexed patterns: 23,090) and (**B**) glucose isomerase (indexed patterns: 7976) delivered in beef tallow. CC (blue circle) and signal-to-noise ratio (red diamond) were plotted as a function of resolution.

For glucose isomerase, 27,380 images were included Bragg peaks, and the X-ray hit rate was 34.22%. Of these, 7795 images were indexed and 181 image showed multi crystal hits. The indexing rate and multi crystal hit rate were 28.47% and 2.32%, respectively. Data were processed at 1.60 Å with 100% completeness, including multiple crystal diffraction patterns. The SNR, R_split_, CC, and CC* of the total data collected were 4.79, 16.70, 0.9656, and 0.9912, respectively. The background scattering of beef tallow delivery medium did not significantly impact the data processing (Fig. 2).

**Figure 3:**
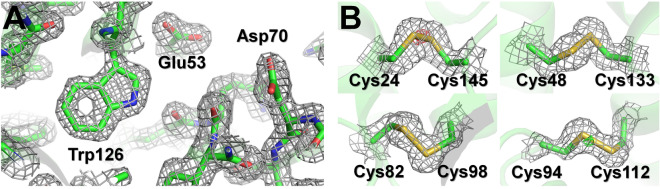
Electron density map of room-temperature lysozyme embedded in beef tallow. (**A**) 2mFo-DFc electron density map (grey mesh, 1.2σ) of lysozyme (**B**) 2mFo-DFc (grey mesh, 1.2σ) and mFo-DFc (green, 3σ; red, − 3σ) electron density map around disulfide bonds of lysozyme.

The final model structure of lysozyme was refined to 1.55 Å, with R_work_ and R_free_ of 16.79 and 18.79%, respectively. The overall electron density map of lysozyme was clearly observed from Lys19 to Leu145 (Fig. 3A). The room-temperature structure of lysozyme embedded in beef tallow revealed higher similarity with other room-temperature structures of lysozyme embedded in polyacrylamide (PDB code 6IG6 and 6JXQ), shortenings (6KCB and 6KCD), agarose (6KD1 and 6LL3), wheat starch (7BVM), alginate (7BVO), and lard (7CJZ), showing an r.m.s. deviation of 0.092–0.221 Å for all Cα atoms. Electron density maps were analyzed for four disulfide bonds (Cys24–Cys145, Cys48–Cys133, Cys82–Cys98, and Cys94–Cys112), which are more sensitive to radiation damage than other areas of the structure. The maps revealed the absence of significant negative peaks, indicating that no substantial radiation damage is present (Fig. 3B).

**Figure 4:**
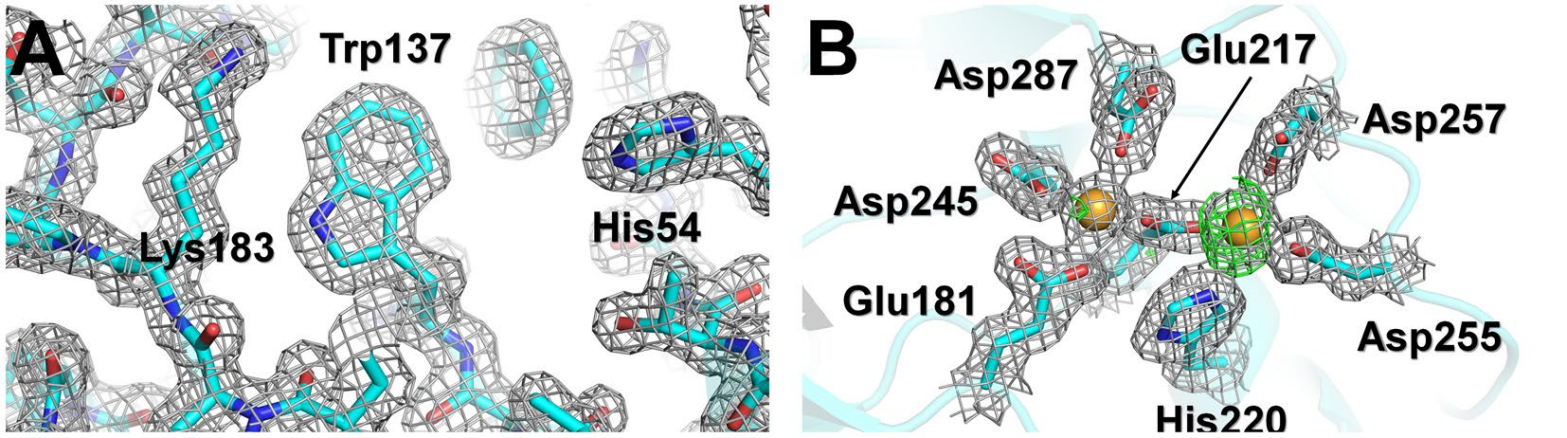
Electron density map of room-temperature glucose isomerase embedded in beef tallow. (**A**) 2 mFo-DFc electron density map (grey mesh, 1.2σ) of glucose isomerase. (**B**) 2mFo-DFc (grey mesh, 1.2σ) and mFo-DFc (green, 3σ; red, − 3σ) electron density map of metal binding sites at the active site of glucose isomerase.

The final model structure of glucose isomerase was refined to 1.60 Å, with R_work_ and R_free_ of 16.81 and 18.62%, respectively. The overall electron density map of glucose isomerase was clearly observed from Tyr3 to Arg387 (Fig. 4A). The room-temperature structure of glucose isomerase embedded in beef tallow was similar to other room-temperature structures of glucose isomerase embedded in shortening (PDB code 6KCA and 6KCC), gelatin (6KD2 and 6LL2), wheat starch (7BVL), and alginate (7BVN), showing an rms deviation of 0.082–0.385 Å for all Cα atoms. The active site of glucose isomerase has two metal binding sites, M1 and M2, which are involved in substrate recognition and isomerase activity, respectively^62–64^. Mg^2+^ or Mn^2+^ can bind to the metal-active site of GI from *Streptomyces rubiginosus* used in this experiment. When the GI model was refined by positioning Mg^2+^ on the M1 and M2 sites, a positive Fo-Fc electron density map of M2 sites was observed (Fig. 4B). In contrast, when Mn^2+^ was positioned at the M2 site and refinement was performed, a negative Fo-Fc electron density map was observed (Supplementary Fig. S3). The metal species or the radiation damage is hypothesized to cause the difference in the Fo-Fc density map of the M2 site. In the first case, a mixture of Mg^2+^ and Mn^2+^ binds to the M2 sites of the numerous GI proteins present in the crystal. Considering that positive and negative Fo-Fc density maps were obtained for Mg^2+^ and Mn^2+^ at the M2 site, respectively, Fo-Fc density maps would not have been observed if the refinement was performed at a specific ratio at which Mn^2+^ binds to a larger number of sites than Mg^2+^. In the second case, radiation damage occurred while the majority of the M2 sites of the GI were occupied by Mn^2+^. The GI M2 site is known to be highly radiation-sensitive, and radiation damage was observed for a diffraction weighted dose (DWD) of 0.7 MGy at the GI M2 site under cryogenic conditions^65^. Furthermore, using room-temperature crystallography, a *D*_1/2_ (the dose required to halve the original diffraction intensity) limit of 0.57 MGy was determined for lysozyme^66^. The average diffracted weight dose for X-rays used in this experiment was calculated to be 0.607264 MGy. Therefore, if Mn^2+^ occupied the M2 site of the GI crystal used in the experiment, radiation damage occurred. However, in this study, the M2 site of the GI used was not analyzed; therefore, whether the difference arises from the ratio of Mn^2+^/Mg^2+^ bound to the M2 site or from radiation damage remains unclear. Nevertheless, when considering radiation damage studies in the future, it is essential to ensure that the duration of X-ray exposure is shorter than that in this experiment to avoid radiation damage.

### Analysis of X-ray background scattering

**Figure 5:**
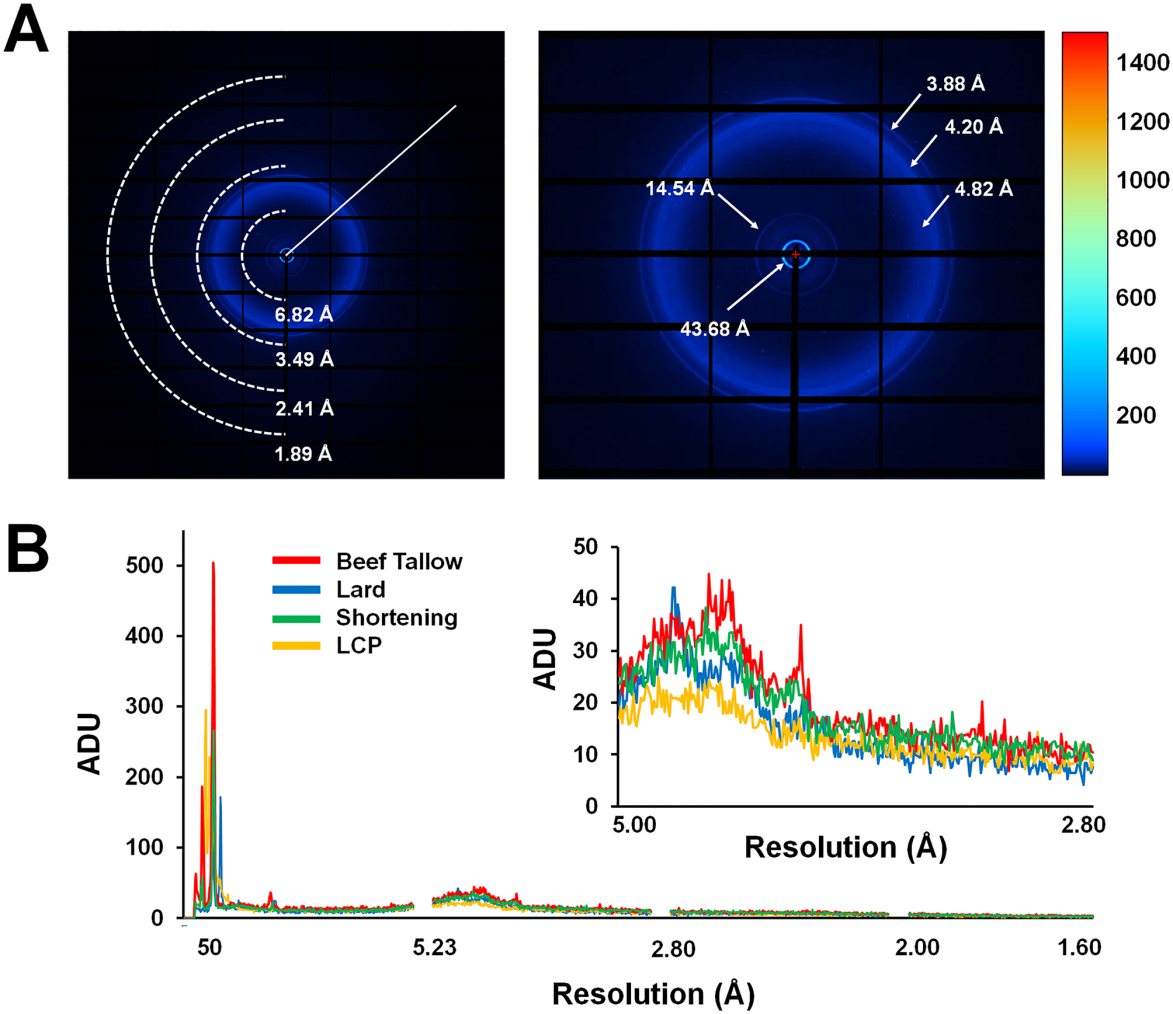
Analysis of X-ray background scattering of beef tallow. (**A**) (Left) Typical scattering image of 80% (v/v) beef tallow. Diagonals in the image on the left are used to generate the intensity plots in (**B**). (Right) Magnification of background scattering of beef tallow from the center of the detector to 3.0 Å in the image on the left. (**B**) Two-dimensional profile of the average scattering intensities of 80% (v/v) beef tallow, 80% (v/v) lard, 80% (v/v) shortening, and 60% (v/v) LCP (monoolein). Inset shows a magnified view of the intensity of background scattering in the 5–2.8 Å resolution range.

In the collected diffraction data, beef tallow exhibited intrinsic X-ray scattering (Fig. 5A). Although such background scattering did not significantly affect the processing of diffraction data of lysozyme and glucose isomerase (Fig. 2 and Table 1), background scattering of beef tallow may affect the SNR of the data. Therefore, it is important to analyze the degree of background scattering at various resolutions to select and apply the appropriate sample delivery medium. Accordingly, background scattering of beef tallow was analyzed and compared with those of fat-based delivery media (shortening and lard) and LCP (60% monoolein), which is widely used as a sample delivery medium for SX data collection. Beef tallow showed background scattering of 504.6, 36.4, 22.5–44.8, and 26.8 analog-to-digital units (ADUs) at 43.68, 14.54, 4.82–4.20 and 3.88 Å, respectively (Fig. 5A). The X-ray scattering patterns in these specific resolutions were likely caused by inherent packing of the interactions among the molecules constituting beef tallow. Shortening showed background scattering of 266.2, 21.8, 23.6–38.4, and 25.4 ADUs at 44.02, 14.32, 5.0–4.20, and 3.90 Å, respectively (Fig. 5B). Lard showed background scattering of 158.8, 172.2, 25.2, 24.6, and 19.4–42.2 ADUs at 45.63, 35.40, 22.35, 13.70, and 5.0–3.8 Å, respectively (Fig. 5B). LCP showed background scattering of 295.2 and 17–23.2 ADUs at 44 Å and 5.0–3.8 Å, respectively (Fig. 5B). Thus, beef tallow shows a relatively high background scattering compared to other sample delivery media but had no significant impact on data processing (Fig. 2). Despite the differences in the scattering intensity, the background scattering of beef tallow is similar to the intrinsic background scattering of lard, which was previously reported. This may be because the main components (oleic, palmitic, and linoleic acids) of these fat-based media are similar^61^. In contrast, the differences in the scattering among the media were considered to result from the different proportions of the constituents of fats.

## Discussion

Previously, shortening and lard were employed as fat-based delivery materials. Their melting temperatures are 26.5–28.5 and 36 °C, respectively. In this study, beef tallow, which has a melting temperature of 42 °C (which is higher than those of shortening and lard) and possesses the advantages of existing fat-based delivery media, was used as the delivery medium for the SX samples.

As beef tallow has a higher melting point than shortening and lard, it has an advantage in two aspects of SX research. Firstly, many proteins and enzymes show higher activity at temperatures higher than room temperature. If the target molecule has a high activity at 37 °C and the crystal lattice remains stable, collecting data at this optimum activity temperature is biologically more significant. In this respect, beef tallow can provide a relatively more stable injection stream in a temperature environment higher than room temperature compared to shortening and lard. Secondly, in time-resolved studies using optical lasers (e.g. ns-laser), the heat generated temporarily when the optical laser passes through the viscous medium may affect the stability of the injection stream. Because beef tallow has a higher melting temperature than shortening or lard, it will have less impact on these instantaneous heat sources. As a result, beef tallow is less affected by temperature or heat than the previously developed shortening or lard injection medium. It is necessary to study the influence of the characteristics of the optical laser on the stability of the injection stream for pump-probe research using beef tallow as the delivery medium. Moreover, in a previous study, shortening and lard showed a stable injection stream even at 100 nL/min^54, 55^, whereas beef tallow generated a much more stable injection stream even at lower flow rates of 10 nL/min. As a result, because the injection stream stability of beef tallow is superior to that of shortening and lard even at a lower flow rate, the crystal sample can be stably extruded from the syringe at a lower flow rate, so that the consumption of the crystal sample can be drastically reduced.

In this experiment, it was demonstrated that beef tallow provides a stable injection stream in the flow rate range of 10–200 nL/min. In fact, the injection stream remains stable at high flow rates of several tens of mL/min as well as low flow rates of 1 nL/min. Therefore, beef tallow has no limitations with respect to flow rates owing to its high viscosity. However, experimentally, if the flow rate is excessively low, crystal samples may be exposed to X-rays more than once, and if the flow rate is excessively high, the sample consumption will increase. Consequently, it is essential to optimize the flow rate of the injection stream based on the characteristics (size and exposure time) and crystal density of X-rays. Beef tallow exhibited background scattering patterns that were distinct from those of other fat-based sample delivery media. The background scattering of beef tallow did not significantly affect the data processing; however, a relatively high background scattering was observed at approximately 45 Å was observed compared with other shortenings and lards. In experiments requiring low-resolution area data, materials can be delivered via an inner diameter smaller than the syringe needle of the 168-µm inner diameter used in this experiment to reduce the background scattering. In this case, sample consumption also can be significantly reduced.

Since beef tallow can absorb light at a broad range of wavelengths (300–700 nm), viscous media can absorb the signal emitted by the excited state in time-resolved SX by optical excitation methods, which should be considered when employing tallow as a medium for time-resolved studies using optical lasers. To minimize the light absorption of beef tallow, the injection diameter needs to be minimized. Additionally, as beef tallow is hydrophobic, the ligand solution may not mix homogeneously with beef tallow or penetrate the crystal embedded in beef tallow, which is a limitation of the mixing-and-injecting method. Therefore, it is essential to verify whether beef tallow is suitable for the target experiment prior to conducting time-resolved SX. Because beef tallow is composed of fat, it is not suitable for crystal samples that have specific interactions with lipids. Moreover, to avoid crystal damage due to non-specific interactions with the beef tallow medium, the crystal sample is embedded in beef tallow and incubated for several hours, followed by observation of the crystal morphology or examination of the stability of the crystal by an X-ray diffraction test. In addition, as beef tallow is hydrophobic, mixing it with membrane protein crystals may alter the hydrophobic environment of the crystals, which may affect the quality of the crystals. Therefore, when selecting beef tallow as a sample delivery medium for membrane protein crystals, it is essential to pre-screen whether the membrane protein crystals are stable in beef tallow.

In conclusion, the beef tallow is a new sample delivery medium applicable to SX. These results provide a proof-of-concept for the development of beef tallow injection matrices for SX.

## Methods

### Preparation of beef tallow injection matrix

The extraction and purification process of beef tallow by heat treatment and phase separation was performed according to a previously described lard extraction method with slight modifications^55^. Fat around beef ribs was obtained from a butcher shop. The fat was placed on a stainless steel plate and heated at > 100 °C for 20 min to extract the tallow. In this process, the tissues were burnt and solid impurities were generated. To remove these, extracted beef tallow with impurities was passed through 50-µm mesh pores before solidification. Next, a phase separation method was performed at room temperature using distilled deionized water (DDW) to remove water-soluble impurities extracted with beef tallow. The extracted fat was bottled with boiled DDW, vortexed, and left at room temperature (23–25 °C) until solidified. The bottle cap was opened and water layer was removed. DDW was added to the solidified beef tallow, mixed, and layered at room temperature. When the beef tallow hardened, the DDW layer was removed. This process was performed five times. The obtained beef tallow was placed on a stainless steel plate and heated to > 100 °C to evaporate residual moisture. The beef tallow solution was then placed in a glass vial and stored at 4 °C.

### Chemical compatibility

Beef tallow (40 μL) and a chemical reagent (10 μL; 5 M NaCl, 4.5 M (NH_4_)_2_SO_4_, 100% PEG400, 50% [w/v] PEG3350, 50% [w/v] PEG8000, 100% MPD) were placed inside a 100 μL syringe, connected using a coupler, and subsequently mixed by moving the plunger back and forth more than 30 times. The syringe containing the mixed solution was connected to a needle having an internal diameter of 167 μm and delivered using a syringe pump at a flow rate of 100 nL/min. For chemical compatibility, the stability of the injection stream of beef tallow was estimated visually by its viscosity.

### UV/Vis spectroscopy

After dissolving beef tallow in hot water inside a glass vial, the beef tallow solution (20, 40, 60, and 100 μL) was placed in a 96-well plate (circular shape; diameter: 6.5 mm) and solidified. The absorption of the beef tallow was measured in the range of 300–700 nm using a Synergy™ microplate reader (BioTek) at 25 °C.

### Crystallization

Lysozyme and glucose isomerase were crystallized as reported previously^52, 55^. Briefly, lysozyme from hen egg white was purchased from Hampton Research (HR7–110, Aliso Viejo, CA, USA). The lysoyzme powder was dissolved in buffer containing 10 mM Tris–HCl (pH 8.0) and 200 mM NaCl, and the final concentration of the lysozyme solution was 50 mg/mL. The lysozyme solution (100 μL) and crystallization solution (100 μL) containing 0.1 M Na-acetate, pH 4.6, 6% (w/v) PEG 8000, and 10% (w/v) NaCl were transferred into a 1.5-mL microcentrifuge and immediately mixed with a vortexer. This mixture was incubated at 22 °C overnight. The size of lysozyme crystals was approximately 30 μm. Glucose isomerase from *Streptomyces rubiginosus* was purchased from Hampton Research (HR7-098). This product was supplied in crystalline form, and thus directly used for SX without further purification and crystallization. The crystal size of glucose isomerase was < 60 μm.

### Embedding crystals in beef tallow

Crystals were embedded in beef tallow as previously described for preparing a shortening delivery system^54^. The crystal suspension (30 µL) was transferred to a 100-µL syringe and left for 10 min in the vertical position. When the crystals had settled in the inner channel of the syringe, the supernatant was removed from the crystal suspension by pushing the syringe plunger. The glass vial containing solidified beef tallow was immersed in hot water (> 100 °C) for 1 min. When the beef tallow completely melted to the liquid phase, 40 μL was aliquoted into a 100-μL syringe and left at room temperature until it had solidified. The syringes containing crystals (10 µL) and beef tallow (40 µL) were connected using a syringe coupler, and then gentle back and forth mixing was performed more than 30 times using a plunger. The mixed samples were transferred to one syringe, and the coupler and partner syringe were removed. A syringe needle with an inner diameter of 168 µm was connected to the syringe containing the sample. To prevent the sample from being exposed to air until the experiment, the tip of the syringe needle was sealed with parafilm and stored at room temperature.

### X-ray data collection

SX experiments were performed using a beef tallow delivery medium at the 11C beamline at Pohang Accelerator Laboratory (Pohang, Korea). The photon flux and X-ray wavelength were 1.3 × 10^12^ photons/s and 0.9795 Å, respectively. The X-ray size at the sample position was approximately 4.5 (vertical) × 8.5 (horizontal) μm^2^ (full-width half maximum). The syringe containing the crystals embedded in beef tallow was installed on the Fusion Touch 100 syringe pump (CHEMYX, Stafford, TX, USA). Crystals embedded in beef tallow were extruded from syringe at a flow rate of 100 nL/min via a syringe pump-based sample delivery method^24^. Crystal samples embedded in beef tallow were exposed to X-rays for 100 ms. Diffraction data were collected at 25 ± 0.5 °C and recorded on a Pilatus 6 M with 10 Hz readout. The injection stream was continuously exposed to X-rays, and the detector was used without the shutter mode.

### Structure determination

Images containing Bragg peaks were filtered using the Cheetah program^67^. Hit images were indexed, integrated, and scaled using the CrystFEL^68^ program. The phasing problem was solved by the molecular replacement method using phase-MR in Phenix^69^ with the crystal structures of lysozyme (PDB code 7CVJ)^31^ and glucose isomerase (PDB code 7CJO)^64^ as the search models. Model building was performed using the COOT^70^ program. Structure refinement was performed using Phenix.refine in PHENIX^71^. The model geometry was validated using MolProbity^72^. Structure figures were generated using the PYMOL program (https://pymol.org/). The structure factors and coordinates have been deposited in the Protein Data Bank (PDB) under accession codes 7E02 (lysozyme delivered in beef tallow) and 7E03 (glucose isomerase delivered in beef tallow). Hit images containing the diffraction pattern and geometry files have been deposited in CXIDB under ID 165 (lysozyme derived in beef tallow) and 166 (glucose isomerase delivered in beef tallow).

### Analysis of background scattering

All viscous media were delivered through a syringe needle with inner diameter of 168 μm. In total, 80% (v/v) beef tallow, 80% (v/v) lard, and 60% (v/v) monoolein were delivered at 25 °C, whereas 80% (v/v) shortening was delivered at 20 °C. To analyze the X-ray background scattering of the viscous media, 20 images were randomly selected from the collected images and the intensity was analyzed using ADXV software (https://www.scripps.edu/tainer/arvai/adxv.html). The background scattering of the viscous medium measured the intensity from the center of the detector to 1.6 Å (Fig. 5a), and the average value of the sum of all intensities was shown.

## Supplementary Information

Supplementary Information 1.

Supplementary Video 1.

Supplementary Video 2.

Supplementary Video 3.

Supplementary Video 4.

Supplementary Video 5.
