# Supplementary material for: Beef tallow injection matrix for serial crystallography
Source: Sci Rep. 2022 Jan 13;12:694. doi: 10.1038/s41598-021-04714-6 (PMC8758675; doi:10.1038/s41598-021-04714-6)
Supplement: Supplementary file 1 — Supplementary Information 1. [file 41598_2021_4714_MOESM1_ESM.docx]

**Supplementary Information**

**Beef tallow injection matrix for serial crystallography**

Ki Hyun Nam^1,2,^*

^1^ Department of Life Sciences, Pohang University of Science and Technology, Pohang, Gyeongbuk 37673, Korea

^2^ Biotech Center, Pohang University of Science and Technology, Pohang, Gyeongbuk 37673, Korea

* Corresponding. structures@postech.ac.kr (K.H.N.)

**Contents**

**Supplementary Figures**

Supplementary Figure S1. Schematic illustrating the preparation of beef tallow.

Supplementary Figure S2. UV-visible analysis of beef tallow.

Supplementary Figure S3. Analysis of the electron density map of GI.

**Supporting Videos**

Supplementary Video 1. Beef tallow injection matrix (flow rate of 200 nl/min)

Supplementary Video 2. Beef tallow injection matrix (flow rate of 200 nl/min)

Supplementary Video 3. Beef tallow injection matrix (flow rate of 100 nl/min)

Supplementary Video 4. Beef tallow injection matrix (flow rate of 50 nl/min)

Supplementary Video 5. Beef tallow injection matrix (flow rate of 10 nl/min)


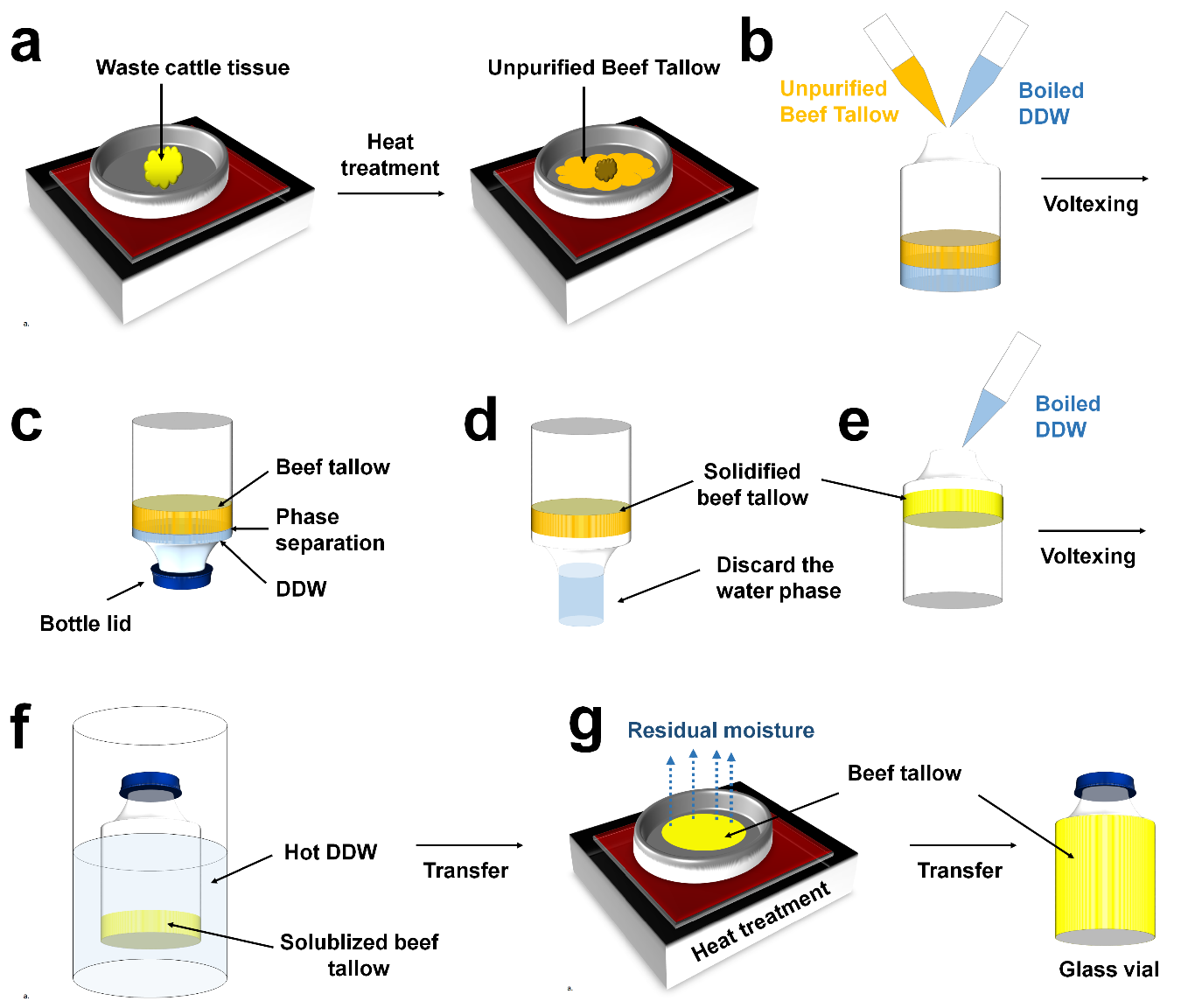


**Supplementary Figure S1. Schematic illustrating the preparation of beef tallow. (**a) Extraction of beef tallow from waste cattle tissue through heat treatment. (b) Beef tallow extract and boiled deionized distilled water (DDW) were transferred into a glass bottle and mixed for 1 minute voltex. (c) The bottle containing the beef tallow and water was stored at room temperature. (d) When the temperature reduced to room temperature and the beef tallow solidified, the lid was opened, and the water was discarded. Steps (c–e) were repeated a minimum of 10 times. (f) Bottle containing the solidified beef tallow was immersed in hot water, and the solublized beef tallow was transferred onto a hot plate. (g) Residual moisture remaining in the beef tallow was removed by heat treatment. Purified beef tallow was transferred into a glass vial and stored at 4 °C.


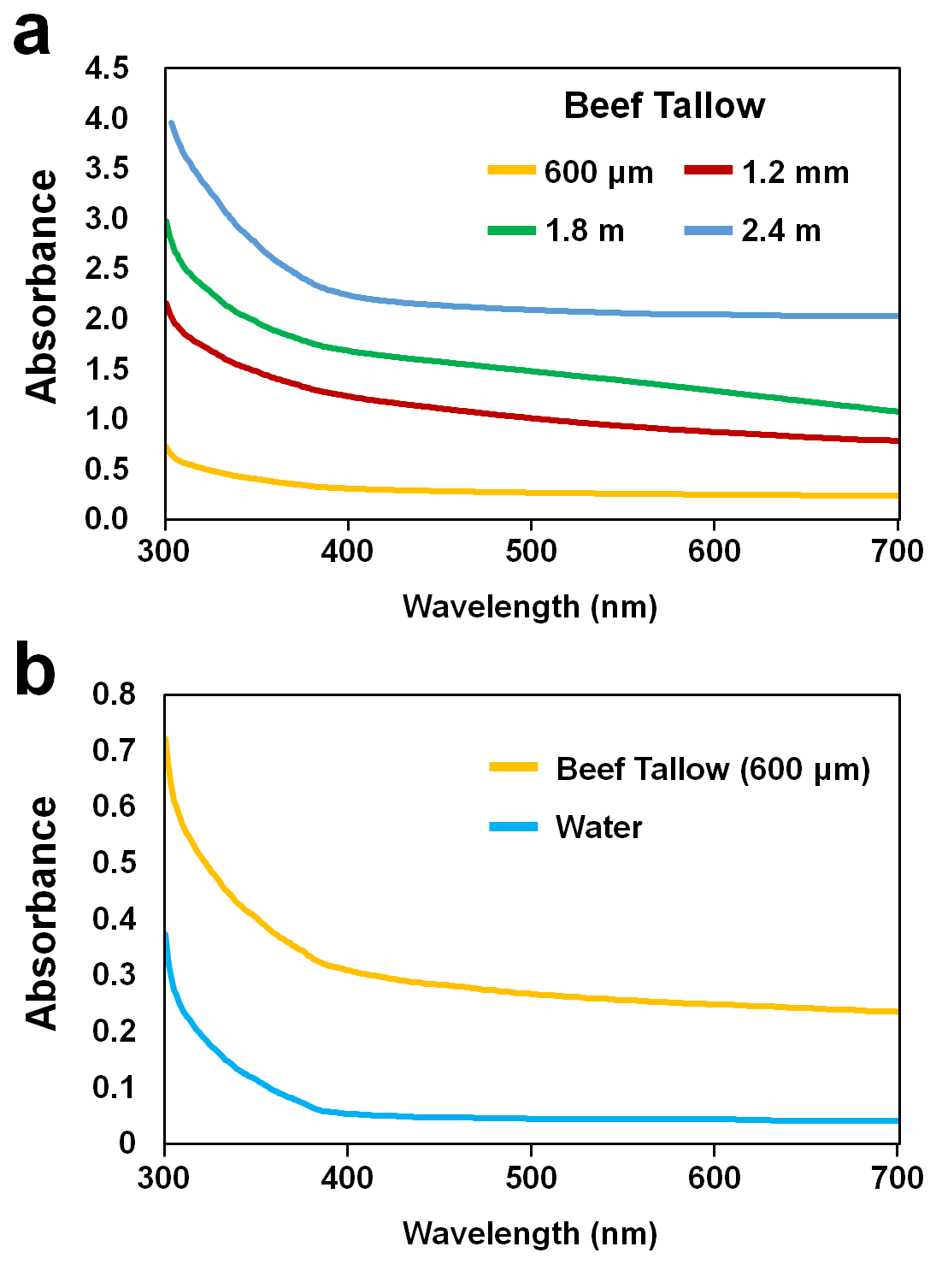


**Supplementary Figure S2.** UV-visible analysis of beef tallow. (a) The absorption spectra of the beef tallow. (a) Absorption spectra of the PLP enzyme (b) Comparison of absorbance spectra between beef tallow (600 μm) and water.


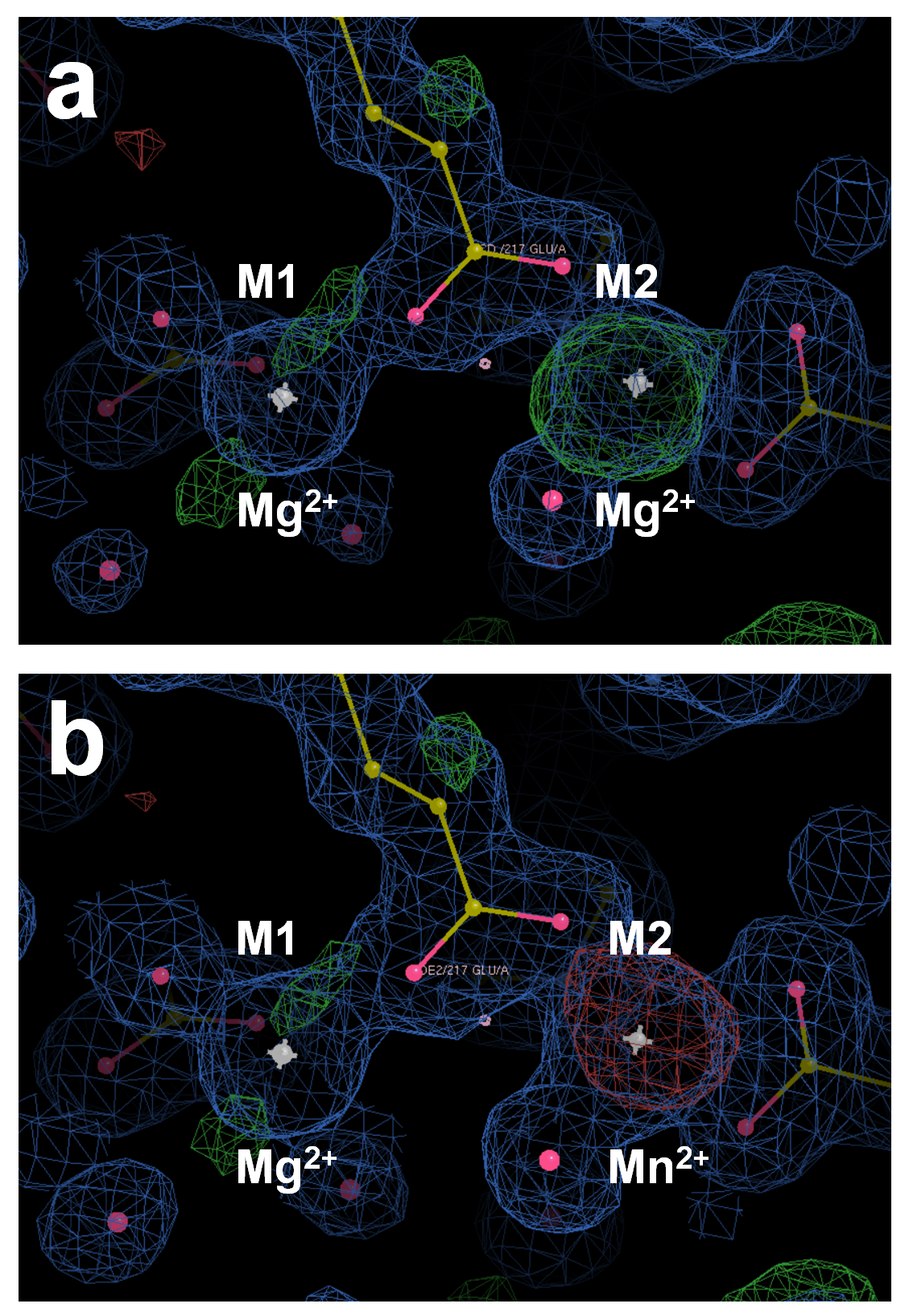


**Supplementary Figure S3.** Analysis of the electron density map of GI. 2mFo-DFc (blue mesh, 1.2σ) and mFo-DFc (green, 3σ; red, -3σ) electron density maps of GI after (a) Mg^2+^ and (b) Mn^2+^ bound to the M2 site.
